# Are Dilatation Instruments Necessary in Gynecological Practice?

**Published:** 1880-08

**Authors:** 


Are Dilatation Instruments Necessary in Gynecological Practice?—This question is asked by Carl Shrœder in No. 26, *Centralblat fur Gynakologie*, 1879, in reference especially to a paper in No. 25 of the same journal by Fritsch, advocating rapid dilatation under chloroform with steel sounds of gradually increasing size, instead of sponge or Sea-tangle tents. Shrœder goes further, and would do away with dilatation in all cases. He proposes, in most cases where dilatation is ordinarily advised, to remove from the interior of the uterus, for examination, a small portion of the mucous membrane through the undilated cervix, by means of a sharp curette with appropriate flexible handle. If found necessary, the hypertrophied mucous membrane may be entirely removed in this way. If it is really necessary to get the finger into the cavity of the uterus, Shrœder proposes to divide on both sides the vaginal portion of the cervix throughout its entire length, and then to pass the finger through the inner os, which in such cases is usually large, by forcible pressure of the index finger. Counter-extension is made either by pressure upon the fundus of the uterus or by traction on the neck with a volsella. The incisions made in the vaginal portion of the cervix Shrœder unites by sutures so soon as the necessary operative interference with the cavity of the uterus is effected. (It appears to us that few people will regard the advantages presented by the heroic methods either of Fritsch or of Shrœder superior to the ordinary method of slow dilatation by the use of tents.—*Edin. Med. Jour.*

